# Interleukin-8 Regulates the Autophagy and Apoptosis in Gastric Cancer Cells via Regulating PI3K/Akt Signaling Pathway

**DOI:** 10.1155/2022/7300987

**Published:** 2022-08-11

**Authors:** Liang Yu, Guoqiang Zhou, Zhiliang Shi, Jian Guo, Shengyuan Yu, Cheng Yu, Chenglong Shen

**Affiliations:** Department of General Surgery, Changshu No. 2 People's Hospital, China

## Abstract

**Objective:**

To explore the role and mechanism of interleukin-8-mediated autophagy regulation of gastric cancer (GC) cells in GC.

**Methods:**

After cell culture, the SGC7901 cell line was separated into the control group and IL-8 (20 ng/mL) group, IL-8 (40 ng/mL) group, and IL-8 (60 ng/mL) group, to verify the effects of the PI3K/Akt signal path on the modulation of autophagy in GC cells. Western blot detected autophagy markers, ATG12-ATG5 complexes, autophagy-associated pathways, and apoptosis-associated factors in GC cells. Transwell was utilized to identify invasion capability.

**Results:**

Compared with the control group, the expression of LC3II, Atg5, ATG7, Beclin1, Bax, C-cas3, C-cas9, P-PI3K, P-Akt, and ATG12-ATG5 was remarkably elevated in the IL-8 (60 ng/mL) group, IL-8 (20 ng/mL) group, and the IL-8 (40 ng/mL) group. The expression of P62 and Bcl-2 in the IL-8 (60 ng/mL) group was also lower than that of the IL-8 (20 ng/mL) group and IL-8 (40 ng/mL) group, in contrast to the controls. The invasive quantity of GC SGC7901 cells in the IL-8 (60 ng/mL) group was also remarkably higher in contrast to the IL-8 (20 ng/mL) and IL-8 (40 ng/mL) groups. The relative expressions of LC3II, Atg5, ATG7, Beclin1, Bax, C-cas3, C-cas9, P-PI3K, P-Akt, and ATG12-ATG5 complex proteins in LY294002 group were considerably elevated. LC3II, Atg5, ATG7, Beclin1, Bax, C-cas3, C-cas9, P-PI3K, P-Akt, and ATG12-ATG5 were decreased in the IL-8 + LY294002 group. The relative expressions of P62 and Bcl-2 proteins in the IL-8 + LY294002 group were remarkably elevated, and the invasion of SGC7901 cells in the IL-8 group was elevated. In contrast to the IL-8 group, the invasion quantity of gastric cancer SGC7901 cells in the IL-8 + LY294002 group was considerably decreased.

**Conclusion:**

IL-8 promotes autophagy and aggression and suppresses apoptosis of GC SGC7901 cells by regulating PI3K/AKT pathway phosphorylation.

## 1. Introduction

Gastric cancer derives from the mucosal epitheliums of the uppermost layer of the gastric wall, which can emerge in different parts of the stomach and is capable of invading various depths and widths of the stomach wall [1]. The prevalence and mortality of GC are high. According to global statistics, the diagnosed rate of gastric cancer ranks fifth, and the mortality rate is third. The incidence of gastric cancer in developing countries ranks first globally [2], which is an urgent problem to be solved. The occurrence and development of gastric cancer are related to genetic factors, environmental factors, personal diet, helicobacter pylori infection, and others [3]. Interleukin-8 (IL-8) is one of the proinflammatory cytokines in the chemokine family [4]. Existing studies have found that IL-8 is associated with inflammatory diseases and exerts a pivotal effect on the pathogenesis of malignant tumors such as oral cancer [5]. IL-8 plays a biological role mainly by binding its receptors CXCR1 and CXCR2 [6]. Research has revealed that a high expressing level of IL-8 is tightly associated with the angiogenesis of GC tissues, invasion and metastases of GC, the malignant fluid status of patients, and prognosis [7]. Autophagy is a process in which cells self-decompose intracellular substances and play an essential role in adapting to environmental changes such as starvation, hypoxia, infection, and injury [8]. Studies on tumors have displayed that autophagy abnormalities exert a pivotal regulatory effect on the development of tumors [9]. In the process of helicobacter pylori inducing the development of GC, it promotes the occurrence and development of GC by inhibiting the degradation of cytotoxic metabolites by autophagolysosomes [10]. The role of IL-8-mediated autophagy regulation of SGC7901 cells in gastric cancer has not been reported. This research intended to unveil the effects and associated causal links of IL-8-mediated autophagy regulation of GC SGC7901 cells via the PI3K/Akt signal path to offer a novel target for the diagnoses and therapy of GC.

## 2. Methods

### 2.1. Cell Culture and Grouping

Human GC SGC7901 lineage cell SGC7901 was acquired from Shanghai Cell Bank, Chinese Academy of Sciences. The human GC SGC7901 lineage cell was cultured with 10% FBS (Gibco, America) and RPM11640 medium (Gino Biopharmaceutical Co., Ltd.) with 1% double-antibody placed in an incubating device under 37°C and 5% CO2. Follow-up experiments are carried out when the cell confluence reaches 70-80%. The concentration of IL-8 (Boaosen Biotechnology Co., Ltd.) is screened by the MTT method, the old medium of human gastric cancer SGC7901 cell line is discarded, and an equal volume of PBS, 20 ng/mL, 40 ng/mL, and 60 ng/mL IL-8 new medium, respectively, control group, IL-8 (20 ng/mL) group, IL-8 (40 ng/mL) group, and IL-8 (60 ng/mL) group, and continued to incubate for 24 h. In order to verify the effects of the PI3K/Akt signal path on the modulation of autophagy in GC SGC7901 cells, they were separated into the control group, IL-8 group, LY294002 group, and IL-8 + LY294002 group; the control group was added the same volume of PBS to the mankind GC SGC7901 lineage cell; the IL-8 group was added with IL-8 at a concentration of 60 ng/mL in the mankind GC SGC7901 lineage cell; the LY294002 group was added with LY294002 at a content of 20 *μ*mol/L (purchased from APEXBIO, USA). IL-8 at a content of 60 ng/mL and LY294002 at 20 *μ*mol/L were supplemented into the IL-8 + LY294002 group.

### 2.2. Western Blot (WB) Was Used to Detect Autophagy Markers, ATG12-ATG5 Complex, Autophagy-Related Pathways, and Apoptosis-Related Factors in SGC7901 GC Cells

The cells in logarithmic growth phase were collected, RIPA protein lysate was added, and the supernatant was collected. The BCA protein concentration assay kit (Beijing KW Century Biotechnology Co., Ltd.) was used to detect the concentration of total protein in the cells. Atg5 (1:1000), ATG7 (1:1000), Beclin1 (1:1000), ATG12 (1:1000), P62 (1:1000), Bax (1:2000), Bcl-2 (1:1000), C-CAS3 (1:1000), C-CAS9 (1: 1000), PI3K (1:1000), Akt (1:1000), p-pi3k (1:1000), p-akt (1:1000), and GAPDH (1:2000) were incubated at room temperature for 1h and then incubated at 4°C overnight. After rinsing with TBST, the target protein was added to the corresponding hrp-labeled secondary antibody ((1:20) 00), at room temperature for 1.5 h, the target protein bands were detected by ECL chemiluminescence method, and the photos were taken. Antibodies were purchased from MBL, Japan. The corresponding HRP-labeled second antibody (R&D systems, 07-1458, 1 : 2000) was added and shaken under R.T. for 1.5 h, and the targeted protein band was identified via ECL chemiluminescence method and photographed. The antibody was purchased from MBL, Japan.

### 2.3. The Invasiveness of GC SGC7901 Cells Was Detected by Transwell

The 1640 intermediary with 10% serum was supplemented into Transwell's lower chamber (Corning Biotechnology Co., LTD.), and digested cell suspension of GASTRIC cancer SGC7901 cells of each group was added into Matrigel's upper chamber, which was cultivated under 37°C in a 5%CO2 cell incubating device. 24 h later, the chamber was removed, fixed with glutaraldehyde, and the chamber was put into 0.1% crystal violet staining and was performed for 20 min. The culture chamber was inverted, and five transmembrane cells from different fields were selected for counting.

### 2.4. Statistical Treatment

GraphPad Prism 9 program was utilized for statistics, measurement data were represented by ($\bar{x}\pm s$), data contrasts between groups were completed by *t* check, data contrasts between several groups were completed by one-way ANOVA, multiple contrasts were carried out via the SNK-q test, and *P* < 0.05 had significance on statistics.

## 3. Results

### 3.1. Effects of IL-8 on Autophagy Markers and Activation of ATG12-ATG5 Ubiquitination System in SGC7901 Cells of GC

**Figure 1:**
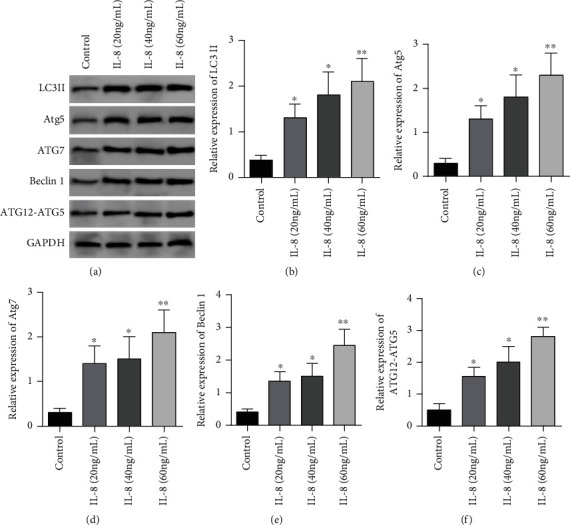
Autophagy markers and ATG12-ATG5 protein expression were affected by different concentrations of IL-8. (a) Western blot was used to detect the effects of different concentrations of IL-8 on autophagy markers and atG12-ATG5 protein expression. (b) Histogram of LC3 ii protein expression. (c) Histogram of Atg5 protein expression. (d) Histogram of ATG7 protein expression. (e) Histogram of Beclin1 protein expression. (f) Protein expression histogram of ATG12-ATG5 complex. ∗*P* < 0.05 compared with the control group, ∗∗*P* < 0.001 in contrast to the controls.

In contrast to the controls, LC3II, Atg5, ATG7, Beclin1, and ATG12-ATG5 complex proteins in IL-8 (20 ng/mL) group, IL-8 (40 ng/mL) group, and IL-8 (60 ng/mL) group. The relative expressing levels were remarkably elevated (*P* < 0.05), the relative expressing levels of LC3II, Atg5, ATG7, Beclin1, and ATG12-ATG5 complex proteins in the IL-8 (60 ng/mL) group were remarkably higher in contrast to the IL-8 (20 ng/mL) group and IL-8 (40 ng/mL) group (*P* < 0.05), see Figure 1.

### 3.2. Roles of IL-8 Incomplete Autophagic Flux in GC SGC7901 Cells

**Figure 2:**
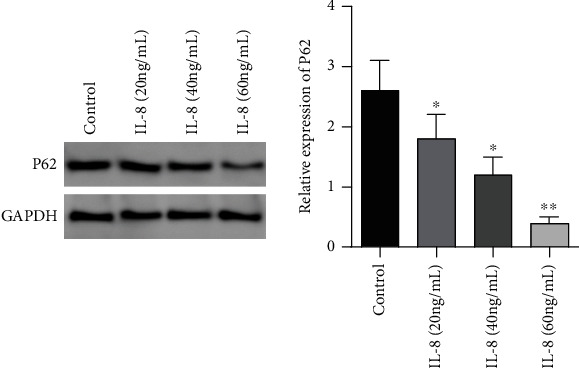
Relative expressing the level of autophagy flux marker protein P62 by different concentrations of IL-8. ∗*P* < 0.05 in contrast to the controls, ∗∗*P* < 0.001 in contrast to the controls.

In contrast to the controls, the expression of autophagy flux marker protein P62 in the IL-8 (20 ng/mL) group, IL-8 (40 ng/mL) group, and IL-8 (60 ng/mL) group was remarkably reduced (*P* < 0.05); the expressing level of autophagy flux marker protein P62 in IL-8 (60 ng/mL) group was remarkably lower in contrast to the IL-8 (20 ng/mL) group and IL-8 (40 ng/mL) group as well (*P* < 0.05), see Figure 2.

### 3.3. The Roles of IL-8 in the Aggression of GC SGC7901 Cells

**Figure 3:**
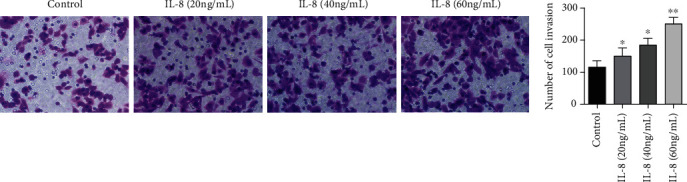
The roles of different concentrations of IL-8 in the invasion number of gastric cancer SGC7901 cells. ∗*P* < 0.05 in contrast to the controls, ∗∗*P* < 0.001 in contrast to the controls.

In contrast to the controls, the invasion number of gastric cancer SGC7901 cells in the IL-8 (20 ng/mL) group, IL-8 (40 ng/mL) group, and IL-8 (60 ng/mL) group was considerably elevated (*P* < 0.05). The invasive quantity of gastric cancer SGC7901 cells in the IL-8 (60 ng/mL) group was also remarkably more remarkable in contrast to the IL-8 (20 ng/mL) group and the IL-8 (40 ng/mL) group (*P* < 0.05), as presented by Figure 3.

### 3.4. Roles of IL-8 in the Expression of Apoptosis-Related Proteins in GC SGC7901 Cells

**Figure 4:**
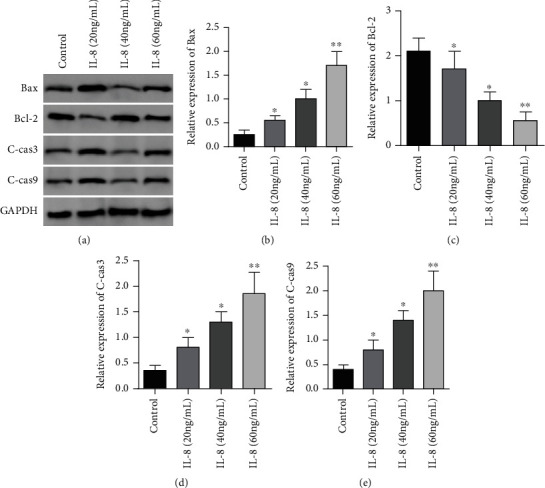
Roles of diverse levels of IL-8 in the expressing of apoptotic proteins in GC SGC7901 cells. (a) Western blot was used to detect the effects of different concentrations of IL-8 on the expression of apoptosis-related proteins in GASTRIC cancer SGC7901 cells. (b) Histogram of Bax protein expression. (c) Histogram of Bcl-2 protein expression. (d) Histogram of c-Cas3 protein expression. (e) Histogram of c-Cas9 protein expression. ∗*P* < 0.05 in contrast to the controls, ∗∗*P* < 0.001 in contrast to the controls.

In contrast to the controls, the expression of Bax, C-cas3, and C-cas9 proteins in GC SGC7901 cells in the IL-8 (20 ng/mL) group, IL-8 (40 ng/mL) group, and IL-8 (60 ng/mL) group. The expressing levels were considerably elevated (*P* < 0.05), and the expression levels of Bax, C-cas3, and C-cas9 proteins in GC SGC7901 cells in the IL-8 (60 ng/mL) group were remarkably higher in contrast to the IL-8 (20 ng/mL) group and IL-8 (40 ng/mL) group as well (*P* < 0.05). In contrast to the controls, IL-8 (20 ng/mL) group, IL-8 (40 ng/mL) group, and IL-8 (60 ng/mL). The expression of Bcl-2 protein in GC SGC7901 cells in the IL-8 (60 ng/mL) group was remarkably lower in contrast to the IL-8 (20 ng/mL) group and IL-8 (40 ng/mL) group as well (*P* < 0.05), see Figure 4.

### 3.5. Effects of IL-8 on Autophagy-Related PI3K/Akt Signal Path in GC SGC7901 Cells

**Figure 5:**
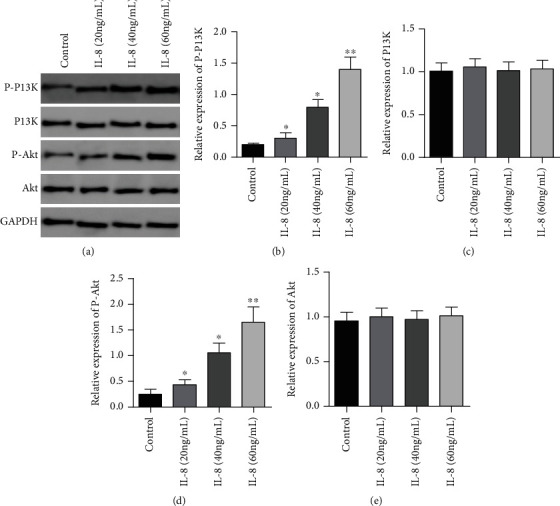
Effects of different concentrations of IL-8 on autophagy-related PI3K/Akt signal path in GC SGC7901 cells. (a) Western blot was used to detect the effects of different concentrations of IL-8 on autophagy-related PI3K/Akt signaling pathway in GASTRIC cancer SGC7901 cells. (b) Histogram of p-PI3K protein expression. (c) Histogram of PI3K protein expression. (d) Histogram of p-Akt protein expression. (e) Histogram of Akt protein expression. ∗*P* < 0.05 in contrast to the controls, ∗∗*P* < 0.001 in contrast to the controls.

In contrast to the controls, the expression of P-PI3K and P-Akt proteins in GC SGC7901 cells in the IL-8(20 ng/mL) group, IL-8(40 ng/mL) group, and IL-8(60 ng/mL) group and the expressing levels of P-PI3K and P-Akt proteins in GC SGC7901 cells in the IL-8 (60 ng/mL) group were remarkably higher in contrast to the IL-8 (20 ng/mL) group and IL-8 (40 ng/mL) group as well (*P* < 0.05), but no remarkable diversity was observed in the expressing of PI3K and Akt proteins in gastric cancer SGC7901 cells in each group (*P* > 0.05), as presented by Figure 5.

### 3.6. Roles of LY294002 in the Expression of Autophagy Marker Genes and Activation of ATG12-ATG5 Ubiquitination System in GC SGC7901 Cells

**Figure 6:**
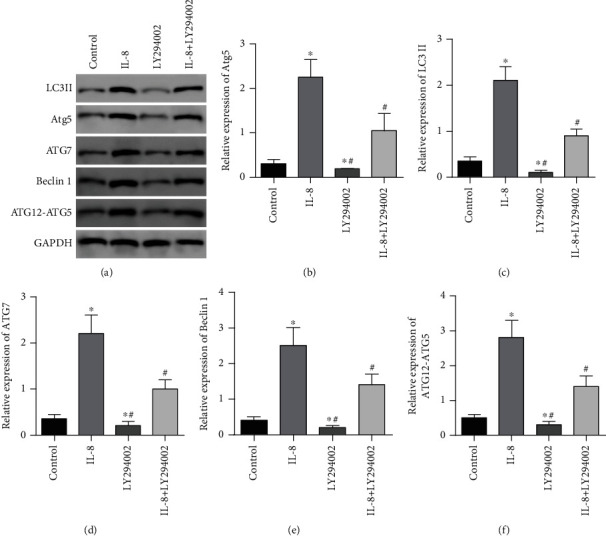
LY294002 on autophagy markers and ATG12-ATG5 protein expression. (a) Western blot was used to detect the effects of LY294002 on autophagy markers and atG12-ATG5 protein expression. (b) Histogram of LC3 II protein expression. (c) Histogram of Atg5 protein expression. (d) Histogram of ATG7 protein expression. (e) Histogram of Beclin1 protein expression. (f) Protein expression histogram of ATG12-ATG5 complex. ∗*P* < 0.05 in contrast to the controls, ^#^*P* < 0.05 in contrast to the IL-8 group.

In contrast to the controls, the relative expressions of LC3II, Atg5, ATG7, Beclin1, and ATG12-ATG5 complex proteins in the IL-8 group were considerably elevated (*P* < 0.05). The relative expression of the level of ATG5 complex protein was considerably diminished (*P* < 0.05). In contrast to the IL-8 group, the relative expression of the level of LC3II, Atg5, ATG7, Beclin1, and ATG12-ATG5 complex proteins in the IL-8 + LY294002 group was remarkably diminished (*P* < 0.05), see Figure 6.

### 3.7. Effects of LY294002 on Complete Autophagic Flux Responses in GC SGC7901 Cells

**Figure 7:**
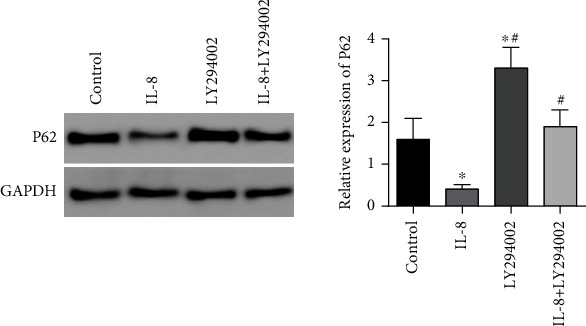
The roles of LY294002 in the complete autophagic flux response of gastric cancer SGC7901 cells. ∗*P* < 0.05 in contrast to the controls, ^#^*P* < 0.05 in contrast to the IL-8 group.

In contrast to the controls, the relative expression of the level of autophagy flux marker protein P62 in the IL-8 group was considerably diminished (*P* < 0.05), and the relative expressing the level of the autophagy flux marker protein P62 in the LY294002 group was remarkably elevated (*P* < 0.05). In contrast to the IL-8 group, the relative expression of autophagy flux marker protein P62 in the IL-8 + LY294002 group was considerably elevated (*P* < 0.05), as presented in Figure 7.

### 3.8. The Effect of LY294002 on the Invasion of GC SGC7901 Cells

**Figure 8:**
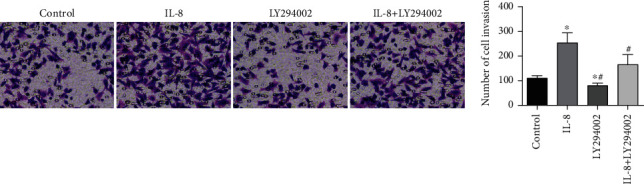
The roles of LY294002 in the invasive quantity of GC SGC7901 cells. ∗*P* < 0.05 in contrast to the controls, ^#^*P* < 0.05 in contrast to the IL-8 group.

In contrast to the controls, the invasion quantity of gastric cancer SGC7901 cells in the IL-8 group was remarkably elevated (*P* < 0.05), and the invasion number of gastric cancer SGC7901 cells in the LY294002 group was remarkably reduced (*P* < 0.05). The invasion quantity of gastric cancer SGC7901 cells in the -8 + LY294002 group was remarkably diminished (*P* < 0.05), as presented in Figure 8.

### 3.9. Roles of LY294002 in the Expression of Apoptotic Proteins in GC SGC7901 Cells

**Figure 9:**
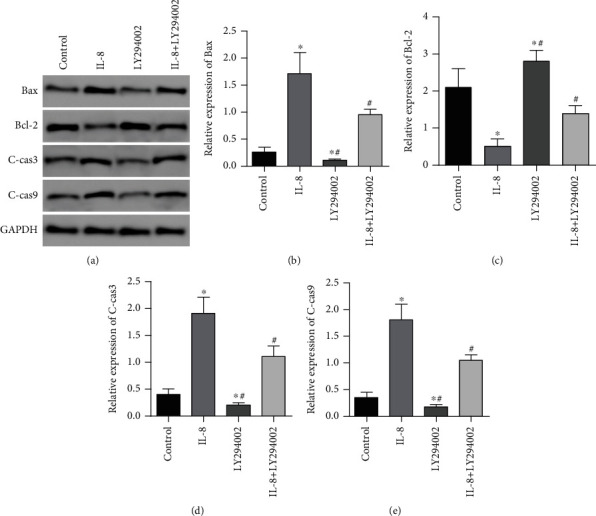
Roles of LY294002 in the expressing apoptotic proteins in GC SGC7901 cells. (a) Western blot was used to detect the effect of LY294002 on apoptosis-related protein expression of GASTRIC cancer SGC7901 cells. (b) Histogram of Bax protein expression. (c) Histogram of Bcl-2 protein expression. (d) Histogram of c-Cas3 protein expression. (e) Histogram of c-Cas9 protein expression. ∗*P* < 0.05 in contrast to the controls, ^#^*P* < 0.05 in contrast to the IL-8 group.

In contrast to the controls, the expressions of Bax, C-cas3, and C-cas9 proteins in the IL-8 group were remarkably elevated (*P* < 0.05). The expressions of Bax, C-cas3, and C-cas9 proteins in the LY294002 group were considerably elevated. In contrast to the IL-8 group, the expressions of Bax, C-cas3, and C-cas9 proteins in the IL-8 + LY294002 group were remarkably diminished (*P* < 0.05). The expression of Bcl-2 protein in the -8 group was remarkably diminished (*P* < 0.05), and the expression of Bcl-2 protein in the LY294002 group was remarkably elevated (*P* < 0.05). In contrast to the IL-8 group, IL-8 + LY294002 expression of Bcl-2 protein in the group was remarkably elevated (*P* < 0.05), as presented in Figure 9.

## 4. Discussion

Gastric cancer ranks first among all cancer types in China, and adenocarcinoma plays a dominant role in gastric malignancies [11]. The symptoms of gastric cancer and precancerous lesions are insidious and non-specific, so early gastric cancer is difficult to find. The pathogenesis of gastric cancer is unknown, which might be associated with various factors, like environmental factors, gene quality, living habits, dietary types, and spiritual factors, as well as persistent gastritis, stomach polyps, gastric mucosal dysplasia, residual stomach after surgery, and long-term helicobacter pylori infection [12]. IL-8 is a glycoprotein with chemotactic and activation effects on neutrophils [13]. Long-term Helicobacter pylori infection will induce gastric mucosal epithelial cells to secrete inflammatory factors such as IL-8, which will collect neutrophils and other neutrophils to the inflammatory site, resulting in gastric mucosal damage and a series of gastrointestinal reactions [14]. Meanwhile, IL-8 is also vital for the angiogenesis of gastric cancer and other malignant tumors [15]. There are many kinds of research about the effects of IL-8 on GC, but there are few reports on the role and mechanism of IL-8-mediated autophagy regulation of SGC7901 cells in gastric cancer.

Autophagy is a conserved intracellular reaction in eukaryotic cells. Its physiological function is to remove aging cells and damaged proteins in cells, maintain the stability of the amino acid library when starving, and regulate innate and adaptive immunity [16]. Dysfunction of autophagy will lead to many illnesses, like infectious diseases, cardiovascular illnesses, neurodegenerative diseases, and aging [17]. Recently, research has revealed that autophagy is tightly associated with the initiation and progression of carcinoma. In some cases, autophagy activation may play a cytotoxic role, thus contributing to eliminating tumor cells [18]. However, it has been stated in some reports that autophagy mainly plays a protective role in cells, which helps maintain the invasiveness and drug resistance of tumor cells [19], indicating that autophagy plays a dual role of inhibition and promotion in the occurrence and development of tumor and can be transformed into each other in some cases.

This study first verified the effect of IL-8 on autophagy in GC SGC7901 cells. The outcomes revealed that with the elevation of IL-8 level, the relative protein-expressing levels of LC3II, Atg5, ATG7, Beclin1, and ATG12-ATG5 complex were also significantly increased. In contrast, the expressing level of autophagy flow marker protein P62 was remarkably decreased. The effects of IL-8 on the invasion and programmed cell death of SGC7901 GC cells were also verified. The outcomes revealed that with the elevation of IL-8 level, the invasion number of SGC7901 gastric cancer cells was considerably elevated, and the expressing levels of apoptotic proteins Bax, C-cas3, and C-cas9 were elevated. In contrast, the expressing level of Bcl-2 protein was considerably reduced. Those outcomes unveil that IL-8 can trigger the autophagy of GC SGC7901 cells and promote the invasion and inhibition of apoptosis of GC SGC7901 cells. PI3K/Akt has been proved to be the main pathway for cells to regulate autophagy and DNA repair [20, 21]. PI3K is one of the critical enzymes in the cell signal transduction pathway. The P13K-mediated signal path exerts a strong regulatory effect on cell growth, proliferative ability, survival, apoptosis, metabolism, and other processes [22]. PI3K is also one of the key molecules in the common autophagy pathway and is vital for the PI3K/Akt autophagy pathway [23]. AKT is a downstream serine/threonine-protein kinase of PI3K as a target protein. It acts as a central enzyme in the cell survival signaling pathway, transmitting extracellular stimulus signals such as growth factors. It is activated and phosphorylated by extracellular factors in a PI3K dependent form [24]. This study verified the effect of IL-8 on the PI3K/Akt signaling pathway, and the outcomes revealed that phosphorylated PI3K and Akt levels elevated remarkably with the increase of IL-8 concentration. In other words, IL-8 enters the nucleus or cytoplasm through phosphorylation to P-PI3K and P-Akt, activating various intracellular enzyme cascade reactions, thus regulating cell apoptosis and autophagy other physiological processes.

In this experiment, IL-8 was used as a treatment factor, combined with LY294002, a specific suppressor of the PI3K/AKT path, to further explore the autophagy ability of IL-8 on gastric cancer SGC7901 cells. The relative expressing levels of LC3II, Atg5, ATG7, Beclin1, and ATG12-ATG5 complex proteins were significantly decreased; the complete autophagic flux response P62 protein was considerably elevated; the number of cell invasions was remarkably diminished; and the Bax, C-cas3, and C-cas9 proteins were considerably decreased. The expression of Bcl-2 protein was significantly decreased, and the expression of Bcl-2 protein was remarkably elevated. This indicates that LY294002 can reduce autophagy and invasion and facilitate the programmed cell death of GC SGC7901 cells by IL-8. Relevant research has unveiled that the expression of IL-8 is high in malignant tissues. H. pylori infection [7]. Studies have shown that IL-8 promotes the invasion and metastasis of various malignant tumors such as gastric cancer [25]. In liver cancer cells, IL-8 can promote liver cancer via the PI3K/AKT signal path. To improve the invasion and metastasis of liver cancer cells [26]. Another study showed that increased IL-8 concentration enhanced the autophagy of neutrophils in patients with rheumatoid arthritis [27].

In conclusion, IL-8 can promote autophagy and invasion and inhibit the apoptosis of SGC7901 GC cells, which may be realized via modulating the phosphorylation of the PI3K/AKT path. The intervention of autophagy-mediated by the PI3K/AKT signal path might be an underlying target for the therapies of GC.

However, this study still has some limitations, and it would be better to add some more animal experiments. This study lacks related clinical research, and it still needs to be further validated in a larger patient cohort.

## Data Availability

The data used to support the findings of this study are available from the corresponding author upon request.
